# Association of potentially functional variants in the *XPG* gene with neuroblastoma risk in a Chinese population

**DOI:** 10.1111/jcmm.12836

**Published:** 2016-03-28

**Authors:** Jing He, Fenghua Wang, Jinhong Zhu, Ruizhong Zhang, Tianyou Yang, Yan Zou, Huimin Xia

**Affiliations:** ^1^ Department of Pediatric SurgeryGuangzhou Women and Children's Medical CenterGuangzhou Medical UniversityGuangzhouGuangdongChina; ^2^ Sun Yat‐sen University Cancer CenterState Key Laboratory of Oncology in South ChinaDepartment of Experimental ResearchCollaborative Innovation Center for Cancer MedicineGuangzhouGuangdongChina; ^3^ Molecular Epidemiology Laboratory and Department of Laboratory MedicineHarbin Medical University Cancer HospitalHarbinHeilongjiangChina

**Keywords:** *XPG*, polymorphism, neuroblastoma, DNA repair, genetic susceptibility

## Abstract

*XPG* gene plays a critical role in the nucleotide excision repair pathway. However, the association between *XPG* gene polymorphisms and neuroblastoma risk has not been investigated. In this study with 256 neuroblastoma cases and 531 cancer‐free controls, we investigated the effects of five potentially functional polymorphisms (rs2094258 C>T, rs751402 C>T, rs2296147 T>C, rs1047768 T>C and rs873601G>A) on neuroblastoma risk. We calculated odds ratio (OR) and 95% confidence interval (CI) to evaluate the association between the five selected polymorphisms and neuroblastoma risk. False‐positive report probability (FPRP) was utilized to determine whether significant findings were noteworthy or because of a chance. We also performed genotype–phenotype association analysis to explore the biological plausibility of our findings. We found that the rs2094258 T allele was significantly associated with decreased neuroblastoma risk (CT *versus* CC: adjusted OR = 0.65, 95% CI = 0.47–0.90, *P* = 0.010; and CT/TT *versus* CC: adjusted OR = 0.71, 95% CI = 0.53–0.97, *P* = 0.030) after adjusting for age and gender. The association was more prominent for subjects with retroperitoneal tumour or early‐stage tumour. We also found that carriers of the 2–3 risk genotypes had a significantly increased neuroblastoma risk when compared to carriers of the 0–1 risk genotypes. The association with risk genotypes was more predominant in older children, females and subjects with retroperitoneal tumour or early stage. Our results were further supported by FPRP analysis and genotype–phenotype association analysis. In conclusion, our study verified that the *XPG* gene rs2094258 C>T polymorphism may contribute to neuroblastoma susceptibility. Our findings require further validation by studies with larger sample size and concerning different ethnicities.

## Introduction

Neuroblastoma has been recognized as the most common childhood extracranial solid tumour, and the third leading cause of tumour‐related death in children 1. The incidence peaks in infancy with a median age at diagnosis of approximately 17 months 2. Despite the utilization of multiple modality treatment involving intensive chemotherapy, radiotherapy and autologous bone marrow transplantation, cure rates for high‐risk patients remain 40% or less. Survivors are frequently subject to serious lifelong coexisting conditions and have poor outcomes 3. With the incidence rate of about 7.7 per million, neuroblastoma is the fourth most frequently diagnosed solid tumour in Chinese children after CNS tumours, lymphomas and germ cell tumours (incidence rate: 23.8, 11.0 and 7.8 per million, respectively) 4. Neuroblastoma is a worldwide public health problem. To date, common environmental exposures that can increase neuroblastoma risk have not been well documented 5, 6. It was suggested that fathers who exposed to some factors (*e.g*., wood dust, solders, radiation sources and hydrocarbons) were more likely to have children with neuroblastoma 5, 6. However, even if fathers were exposed to the same risk environment, only a small proportion of their offspring finally developed neuroblastoma, indicating that genetic factors (*e.g*., polymorphism) may also contribute to neuroblastoma 6.

Genome‐wide association studies (GWASs) have provided evidence that common genetic variants are associated with neuroblastoma susceptibility, with several neuroblastoma susceptibility loci identified 7, 8, 9, 10, 11. In the first GWAS for neuroblastoma, a total of 1032 neuroblastoma patients and 2043 controls of European descent were genotyped by using Illumina HumanHap550 BeadChip covering approximately 500,000 single nucleotide polymorphisms (SNPs). Sequentially, an addition of 720 patients and 2128 controls were tested to further validate significant SNPs identified using first‐stage data. The two‐stage GWAS discovered that three SNPs (rs6939340 A>G, rs4712653 T>C and rs9295536 C>A) within the *CASC15* gene at 6p22 were associated with neuroblastoma susceptibility 7. The association between these three SNPs and neuroblastoma susceptibility has been verified by studies with different ethnicities, such as African–Americans 12, Italians 13, Northern Chinese population 14 and Southern Chinese children 15. We previously confirmed the association of the three SNPs with decreased neuroblastoma risk in Southern Chinese children with a total of 201 neuroblastoma cases and 531 cancer‐free controls 15. GWASs serve as a powerful and important tool to discover inherited common genetic variations for human diseases including cancer 16, 17. It can identify genes previously not implicated in cancer susceptibility by utilizing an agnostic approach. Thus, because of the adoption of restricted *P*‐value (1 × 10^−5^), most of the GWAS‐identified SNPs only have modest risk effects, usually with odds ratio (OR) ranging from 1.1 to 1.5 17. Moreover, the majority of them were located in the intron regions without function. Polymorphisms in the candidate genes, especially functional SNPs, represent a more meaningful way to investigate the role of genes in cancer risk. Previous studies have identified several neuroblastoma risk loci in Chinese populations, such as functional polymorphisms in the *FAS*/*FASL* system genes 18 and *TGFBR3L* gene 19.

DNA repair genes play critical roles in maintaining the integrity and stability of genomic DNA, and about 130 genes have been reported to be involved in the five major DNA repair pathways, including nucleotide excision repair (NER) pathway 20. The NER pathway are mainly responsible for the removing of DNA adducts and lesions 21. In humans, xeroderma pigmentosum group G/excision repair cross‐complementation group 5 (XPG/ERCC5, MIM:133530) is a member of the xeroderma pigmentosum complementation groups (XPA, XPB, XPC, XPD, XPE, XPF and XPG) 22. It functions as an endonuclease and cuts the damaged DNA at the 3′ ends of lesions during the process of DNA repair 23. Thus far, investigations for the association between *XPG* gene polymorphisms and cancer susceptibility have mainly focused on rs17655G>C polymorphism (Asp1104His) and conclusions were controversial 24. The associations of potentially functional polymorphisms in the *XPG* gene with neuroblastoma susceptibility have not been investigated yet. With these in mind, we performed this study including 256 neuroblastoma cases and 531 cancer‐free controls from Southern Chinese children to investigate the association between five potentially functional polymorphisms (rs2094258 C>T, rs751402 C>T, rs2296147 T>C, rs1047768 T>C and rs873601G>A) and neuroblastoma susceptibility.

## Materials and methods

### Study subjects

The current retrospective hospital‐based case–control study consisted of 256 patients with newly diagnosed and histopathologically confirmed neuroblastoma. Patients were enrolled from the Department of Pediatric Surgery, Guangzhou Women and Children's Medical Center, mainly between February 2010 and November 2015. We also recruited 531 age‐, gender‐, and ethnicity‐matched cancer‐free controls from the same geographical regions (Table S1) as described previously 15, 25. Written informed consent was obtained from each subject or his/her guardian. The response rate was about 90% for neuroblastoma subjects and 95% for cancer‐free controls. The study protocol was approved by the Institutional Review Board of Guangzhou Women and Children's Medical Center.

### SNP selection and genotyping

As shown in Table S2, we chose five potentially functional *XPG* SNPs (located in the 5′‐ flanking region, 5′ UTR, exon and 3′ UTR) in this study. Of them, three (rs2094258 C>T, rs2296147 T>C and rs873601G>A) were reported as potentially functional SNPs in a previous investigation 26. The rs751402 C>T polymorphism located in the 5′ UTR was also reported in another study 27. Besides, we chose rs1047768 T>C since it can lead to splicing alteration as predicted by online website SNPinfo (http://snpinfo.niehs.nih.gov/). The widely studied rs17655G>C polymorphism was not included in this study because of strong evidence of linkage disequilibrium between this SNP and rs873601G>A polymorphism (*R* ^2^ = 0.91). All of these chosen polymorphisms had a minor allele frequency >5% in Chinese Han subjects. Single nucleotide polymorphism genotyping was performed in 384‐well plates using Taqman methodology as described elsewhere 15, 26.

### Genotype–phenotype correlation analysis

We performed genotype–phenotype correlation analysis in order to provide biological evidence for our findings. We explored the effects of *XPG* SNPs on gene expression by evaluating the correlation between genotype data of 270 individuals from HapMap phase II release 23 data set containing genotypes of 3.96 million SNPs from four populations (http://hapmap.ncbi.nlm.nih.gov/), and corresponding *XPG* gene transcripts from EBV‐transformed B lymphoblastoid cell lines of the same 270 subjects from SNPexp (http://app3.titan.uio.no/biotools/help.php?app5snpexp). The four populations were as follows: CEU: 90 Utah residents with ancestry from northern and western Europe; CHB: 45 unrelated Han Chinese in Beijing; JPT: 45 unrelated Japanese in Tokyo; YRI: 90 Yoruba in Ibadan, Nigeria. For more details on the study subjects and methodology, refer to previous publications 26, 28, 29.

### Statistical analysis

Distributions of demographic variables and genotype frequencies in neuroblastoma cases and controls were evaluated using chi‐squared test. Hardy–Weinberg equilibrium (HWE) was calculated for controls using the goodness‐of‐chi‐squared test. Odds ratios and corresponding 95% confidence intervals (CIs) were used to estimate the association between selected polymorphisms and neuroblastoma risk. Adjusted ORs were calculated by multivariate analysis with unconditional logistic regression, with adjustment for age and gender. We also computed the false‐positive report probability (FPRP) values for the significant findings. We set 0.2 as FPRP threshold and adopt a prior probability of 0.1 to detect OR of 1.50/0.67 (risk/protective effects) as described previously 30, 31. The association that reached the FPRP threshold of <0.2 was considered noteworthy. All statistical tests were two‐sided, with a significance level of *P* < 0.05. All statistical analyses were performed using SAS software (Version 9.1; SAS Institute, Cary, NC, USA).

## Results

### Demographic characteristics of the subjects

Demographic characteristics of the neuroblastoma cases and cancer‐free controls were presented in Table S1. We included 256 neuroblastoma patients and 531 cancer‐free controls in this study. There were no significant differences in age (*P* = 0.239) and gender (*P* = 0.333) between the two groups. According to the INSS criteria 32, there were 54 (21.09%), 65 (25.39%), 44 (17.19%), 77 (30.08) and 9 (3.52%) patients with clinical I, II, III, IV and 4s neuroblastoma, respectively. In term of site, 46 (17.97%) neuroblastoma occurred in the adrenal gland, 87 (33.98%) in the retroperitoneal and 90 (35.16%) in the mediastinum.

### Associations between XPG gene polymorphisms and neuroblastoma risk

Of the enrolled subjects, 248 neuroblastoma cases and 531 cancer‐free controls were successfully genotyped. The allele and genotype frequencies of the selected SNPs and their associations with neuroblastoma risk are summarized in Table 1. All of the genotype distributions for the five selected polymorphisms were consistent with the HWE (*P* = 0.701 for rs2094258 C>T, *P* = 0.380 for rs751402 C>T, *P* = 0.583 for rs2296147 T>C, *P* = 0.409 for rs1047768 T>C and *P* = 0.686 for rs873601G>A) in control subjects. When compared with the rs2094258 CC genotype, the variant CT and CT/TT genotypes were significantly associated with decreased neuroblastoma risk (CT *versus* CC: adjusted OR = 0.65, 95% CI = 0.47–0.90, *P* = 0.010; and CT/TT *versus* CC: adjusted OR = 0.71, 95% CI = 0.53–0.97, *P* = 0.030) after adjusting for age and gender. We also observed a borderline statistically significantly increased neuroblastoma risk in the carriers of the rs1047768 C allele (CC *versus* TT: adjusted OR = 1.73, 95% CI = 0.93–3.21, *P* = 0.083; and CC *versus* TT/CT: adjusted OR = 1.68, 95% CI = 0.92–3.08, *P* = 0.092). However, there was no significant association or trend observed between the rest of the three polymorphisms and neuroblastoma risk (Table 1). Compared to the subjects without risk genotype, we found a significant trend toward increased risk for subjects carrying 0–3 risk genotypes (adjusted OR = 1.29, 95% CI = 1.06–1.56, *P* = 0.011). We also observed carriers of 2–3 risk genotypes were significantly more predisposed to developing neuroblastoma when compared to carriers of 0–1 risk genotypes (adjusted OR = 1.47, 95% CI = 1.08–1.99, *P* = 0.013).

**Table 1:** Logistic regression analysis of the association between the five polymorphisms in *XPG* gene and neuroblastoma susceptibility

Genotype	Cases (*N* = 248)	Controls (*N* = 531)	*P* *	Crude OR (95% CI)	*P*	Adjusted OR (95% CI)†	*P* †
rs2094258 C>T (HWE = 0.701)							
CC	116 (46.77)	203 (38.23)		1.00		1.00	
CT	93 (37.50)	254 (47.83)		**0.64 (0.46–0.89)**	**0.008**	**0.65 (0.47–0.90)**	**0.010**
TT	39 (15.73)	74 (13.94)		0.92 (0.59–1.45)	0.725	0.94 (0.60–1.47)	0.770
Additive			0.024	0.87 (0.70–1.08)	0.208	0.88 (0.70–1.09)	0.237
Dominant	132 (53.23)	328 (61.77)	0.024	**0.70 (0.52–0.96)**	**0.024**	**0.71 (0.53–0.97)**	**0.030**
Recessive	209 (84.27)	457 (86.06)	0.509	1.15 (0.76–1.76)	0.509	1.16 (0.76–1.77)	0.482
rs751402 C>T (HWE = 0.380)							
CC	96 (38.71)	208 (39.17)		1.00		1.00	
CT	114 (45.97)	241 (45.39)		1.03 (0.74–1.42)	0.883	1.02 (0.73–1.41)	0.922
TT	38 (15.32)	82 (15.44)		1.00 (0.64–1.58)	0.986	0.99 (0.63–1.56)	0.969
Additive			0.988	1.01 (0.81–1.25)	0.949	1.00 (0.81–1.24)	1.000
Dominant	152 (61.29)	323 (60.83)	0.902	1.02 (0.75–1.39)	0.902	1.01 (0.74–1.38)	0.950
Recessive	210 (84.68)	449 (84.56)	0.966	0.99 (0.65–1.51)	0.966	0.98 (0.65–1.49)	0.933
rs2296147 T>C (HWE = 0.583)							
TT	160 (64.52)	343 (64.60)		1.00		1.00	
CT	79 (31.85)	170 (32.02)		1.00 (0.72–1.38)	0.982	0.99 (0.71–1.37)	0.950
CC	9 (3.63)	18 (3.39)		1.07 (0.47–2.44)	0.867	1.08 (0.47–2.45)	0.860
Additive			0.985	1.01 (0.77–1.33)	0.940	1.01 (0.77–1.32)	0.960
Dominant	88 (35.48)	188 (35.40)	0.983	1.00 (0.73–1.38)	0.983	1.00 (0.73–1.37)	0.990
Recessive	239 (96.37)	513 (96.61)	0.865	1.07 (0.48–2.43)	0.863	1.08 (0.48–2.44)	0.853
rs1047768 T>C (HWE = 0.409)							
TT	135 (54.44)	307 (57.82)		1.00		1.00	
CT	93 (37.50)	198 (37.29)		1.07 (0.78–1.47)	0.685	1.07 (0.78–1.47)	0.679
CC	20 (8.06)	26 (4.90)		1.75 (0.94–3.24)	0.076	1.73 (0.93–3.21)	0.083
Additive			0.200	1.19 (0.93–1.52)	0.161	1.19 (0.93–1.52)	0.168
Dominant	113 (45.56)	224 (42.18)	0.375	1.15 (0.85–1.55)	0.375	1.15 (0.85–1.55)	0.378
Recessive	228 (91.94)	505 (95.10)	0.081	1.70 (0.93–3.12)	0.084	1.68 (0.92–3.08)	0.092
rs873601G>A (HWE = 0.686)							
GG	70 (28.23)	137 (25.80)		1.00		1.00	
AG	112 (45.16)	270 (50.85)		0.81 (0.57–1.17)	0.260	0.82 (0.57–1.18)	0.276
AA	66 (26.61)	124 (23.35)		1.04 (0.69–1.58)	0.847	1.07 (0.70–1.62)	0.767
Additive			0.329	1.02 (0.82–1.26)	0.879	1.03 (0.83–1.27)	0.803
Dominant	178 (71.77)	394 (74.20)	0.475	0.88 (0.63–1.24)	0.475	0.89 (0.64–1.26)	0.518
Recessive	182 (73.39)	407 (76.65)	0.324	1.19 (0.84–1.68)	0.324	1.21 (0.86–1.72)	0.279
Combined effect of risk genotypes							
0	27 (10.89)	82 (15.44)		1.00		1.00	
1	95 (38.31)	231 (43.50)		1.25 (0.76–2.05)	0.380	1.25 (0.76–2.06)	0.375
2	110 (44.35)	191 (35.97)		**1.75 (1.07–2.87)**	**0.027**	**1.73 (1.06–2.84)**	**0.030**
3	16 (6.45)	27 (5.08)		1.80 (0.85–3.83)	0.128	1.81 (0.85–3.86)	0.125
Trend			0.010	**1.29 (1.06–1.57)**	**0.010**	**1.29 (1.06–1.56)**	**0.011**
0–1	122 (49.19)	313 (58.95)		1.00		1.00	
2–3	126 (50.81)	218 (41.05)	0.011	**1.48 (1.10–2.01)**	**0.011**	**1.47 (1.08–1.99)**	**0.013**

The significant results were in bold, if the 95% CI excluded 1 or *P* <0.05. *Chi‐squared test for genotype distributions between neuroblastoma patients and controls. ^†^Adjusted for age and gender.

### Stratified analysis

We performed subgroup analyses by age, gender, tumour sites of origin and clinical stages to evaluate the effects of rs2094258 C>T polymorphism and combined risk genotypes on the risk of neuroblastoma (Table 2). We found that the rs2094258 CT/TT genotypes were significantly associated with a decreased neuroblastoma risk in subjects with tumour in the retroperitoneal (adjusted OR = 0.54, 95% CI = 0.33–0.86, *P* = 0.010) and subjects with early‐stage tumour (adjusted OR = 0.55, 95% CI = 0.37–0.81, *P* = 0.003). When the risk genotypes were combined, the significant association with 2–3 risk genotypes were observed in older children (adjusted OR = 1.49, 95% CI = 1.003–2.21, *P* = 0.049), girls (adjusted OR = 1.74, 95% CI = 1.08–2.79, *P* = 0.023), subjects with retroperitoneal tumour (adjusted OR = 2.13, 95% CI = 1.32–3.45, *P* = 0.002), and those with early‐stage tumour (adjusted OR = 2.11, 95% CI = 1.42–3.15, *P* = 0.0003).

**Table 2:** Stratification analysis of the *XPG* rs2094258 C>T polymorphism and combined risk genotypes with neuroblastoma susceptibility

Variables	rs2094258 (cases/controls)		OR (95% CI)	*P*	Adjusted OR* (95% CI)	*P* *	Combined		OR (95% CI)	*P*	Adjusted OR* (95% CI)	*P* *
	CC	CT/TT					0–1	2–3				
Age, month												
≤18	44/86	53/147	0.71 (0.44–1.14)	0.153	0.71 (0.44–1.14)	0.155	48/137	49/96	1.46 (0.91–2.35)	0.121	1.45 (0.90–2.34)	0.126
>18	72/117	79/181	0.71 (0.48–1.05)	0.088	0.72 (0.48–1.06)	0.098	74/176	77/122	**1.50 (1.01–2.23)**	**0.043**	**1.49 (1.003–2.21)**	**0.049**
Gender												
Females	45/81	55/152	0.65 (0.40–1.05)	0.078	0.65 (0.41–1.05)	0.081	50/148	50/85	**1.74 (1.08–2.80)**	**0.022**	**1.74 (1.08–2.79)**	**0.023**
Males	71/122	77/176	0.75 (0.51–1.12)	0.159	0.76 (0.51–1.13)	0.169	72/165	76/133	1.31 (0.88–1.94)	0.181	1.31 (0.88–1.94)	0.187
Sites of origin												
Adrenal gland	23/203	23/328	0.62 (0.34–1.13)	0.119	0.65 (0.36–1.20)	0.172	23/313	23/218	1.44 (0.79–2.63)	0.240	1.37 (0.75–2.51)	0.311
Retroperitoneal	44/203	37/328	**0.52 (0.33–0.83)**	**0.007**	**0.54 (0.33–0.86)**	**0.010**	32/313	49/218	**2.20 (1.36–3.55)**	**0.001**	**2.13 (1.32–3.45)**	**0.002**
Mediastinum	36/203	53/328	0.91 (0.58–1.44)	0.691	0.89 (0.56–1.42)	0.632	47/313	42/218	1.28 (0.82–2.01)	0.278	1.31 (0.83–2.06)	0.241
Others	9/203	15/328	1.03 (0.44–2.40)	0.943	1.02 (0.44–2.37)	0.970	16/313	8/218	0.72 (0.30–1.71)	0.453	0.71 (0.30–1.70)	0.442
Clinical stage												
I+II+4s	65/203	58/328	**0.55 (0.37–0.82)**	**0.003**	**0.55 (0.37–0.81)**	**0.003**	50/313	73/218	**2.10 (1.41–3.13)**	**0.0003**	**2.11 (1.42–3.15)**	**0.0003**
III+IV	46/203	70/328	0.94 (0.62–1.42)	0.775	1.00 (0.66–1.52)	0.984	67/313	49/218	1.06 (0.70–1.58)	0.814	1.00 (0.66–1.51)	0.998

The significant results were in bold, if the 95% CI excluded 1 or *P* <0.05. *Adjusted for age and gender in logistic regress models.

As shown in Table 3, at the prior probability level of 0.1 and FPRP threshold of 0.2, the association between *XPG* rs2094258 CT genotype and neuroblastoma risk remained noteworthy (FPRP = 0.136). Regarding stratified analysis, the significant increase in neuroblastoma risk for carrier of CT/TT genotypes was noteworthy in the subgroup with early‐stage tumour (FPRP = 0.186). Moreover, in the combined analysis, the associations between 2 and 3 risk genotypes and neuroblastoma risk also reached the FPRP threshold of <0.2, and were considered deserving of attention: the association in the whole study population (FPRP = 0.154), the association in the subgroup with retroperitoneal tumour (FPRP = 0.127) and subgroup with early‐stage tumour (FPRP = 0.043).

**Table 3:** False‐positive report probability analysis for the significant associations between neuroblastoma susceptibility and the rs2094258 C>T and combined risk genotypes of the *XPG* gene

Genotype	Crude OR (95% CI)	*P* *	Statistical power†	Prior probability				
				0.25	0.1	0.01	0.001	0.0001
*XPG* rs2094258 C>T								
CT *versus* CC	0.64 (0.46–0.89)	0.008	0.463	0.050	**0.136**	0.634	0.946	0.994
CT/TT *versus* CC	0.70 (0.52–0.96)	0.024	0.627	0.103	0.255	0.791	0.974	0.997
CT/TT *versus* CC								
Retroperitoneal	0.52 (0.33–0.83)	0.007	0.110	0.153	0.351	0.856	0.984	0.998
Stage I+II+4s	0.55 (0.37–0.82)	0.003	0.126	0.071	**0.186**	0.716	0.962	0.996
Risk genotypes								
2–3 *versus* 0–1	1.48 (1.10–2.01)	0.011	0.530	0.057	**0.154**	0.666	0.953	0.995
>18	1.49 (1.003–2.21)	0.049	0.500	0.206	0.437	0.895	0.989	0.999
Females	1.74 (1.08–2.80)	0.022	0.271	0.195	0.421	0.889	0.988	0.999
Retroperitoneal	2.20 (1.36–3.55)	0.001	0.074	0.046	**0.127**	0.615	0.942	0.994
Stage I+II+4s	2.10 (1.41–3.13)	0.0003	0.060	0.015	**0.043**	0.333	0.834	0.981

The significant results were in bold, if the 95% CI excluded 1 or *P* <0.05. *Chi‐squared test was used to calculate the genotype frequency distributions. ^†^Statistical power was calculated using the number of observations in the subgroup and the OR and *P*‐values in this table.

### Genotype‐based mRNA expression analysis

Results of the genotype‐based *XPG* mRNA expression analysis for rs2094258 C>T and rs1047768 T>C polymorphisms were shown in Table 4. We found the *XPG* mRNA expression levels in rs2094258 CT genotype carriers were significantly elevated when compared to the CC genotype carriers in Africans (*P* = 0.004) and Chinese subjects (*P* = 0.044). We also found a higher *XPG* mRNA expression for rs2094258 CT/TT genotype carriers for Africans (*P* = 0.005) and Chinese subjects (*P* = 0.027). For all subjects, we found a trend toward increased *XPG* mRNA expression (*P* = 0.074). These results were consistent with our findings from the association study. The decreased neuroblastoma susceptibility might be partially attributed to the decrease in *XPG* mRNA expression levels. As to the rs1047768 T>C polymorphism, we only found significantly higher expression in the CC and CT/CC genotype carriers than in the TT genotype carriers for all the mixed subjects (*P* = 0.029 and *P* = 0.040, respectively).

**Table 4:** *XPG* mRNA expression by the genotypes of rs2094258 C>T and rs1047768 T>C, using genotype data from the HapMap (http://hapmap.ncbi.nlm.nih.gov/)* and mRNA expression data from SNPexp (http://app3.titan.uio.no/biotools/help.php?app5snpexp)

Population	rs2094258 C>T					rs1047768 T>C				
	Genotypes	No.	Mean ± S.D.	*P* †	*P*_trend_ ‡	Genotypes	No.	Mean ± S.D.	*P* †	*P*_trend_ ‡
CEU	CC	56	9.69 ± 0.22		0.701	TT	19	9.65 ± 0.29		0.617
	CT	29	9.69 ± 0.24	0.897		TC	44	9.69 ± 0.21	0.541	
	TT	5	9.79 ± 0.33	0.415		CC	27	9.72 ± 0.22	0.370	
	Dominant	34	9.70 ± 0.25	0.889		Dominant	71	9.71 ± 0.21	0.486	
	Recessive	85	9.69 ± 0.22	0.403		Recessive	63	9.68 ± 0.24	0.449	
YRI	CC	61	9.79 ± 0.15		**0.016**	TT	6	9.73 ± 0.10		0.320
	CT	27	9.90 ± 0.19	**0.004**		TC	40	9.83 ± 0.19	0.226	
	TT	2	9.83 ± 0.13	0.757		CC	44	9.84 ± 0.15	0.087	
	Dominant	29	9.90 ± 0.19	**0.005**		Dominant	84	9.83 ± 0.17	0.140	
	Recessive	88	9.83 ± 0.17	0.994		Recessive	46	9.82 ± 0.18	0.480	
CHB	CC	19	9.77 ± 0.21		**0.027**	TT	25	9.84 ± 0.21		0.850
	CT	23	9.90 ± 0.21	**0.044**		TC	16	9.87 ± 0.23	0.674	
	TT	3	9.96 ± 0.16	0.143		CC	4	9.81 ± 0.15	0.785	
	Dominant	26	9.91 ± 0.20	**0.027**		Dominant	20	9.86 ± 0.22	0.784	
	Recessive	42	9.84 ± 0.21	0.349		Recessive	41	9.85 ± 0.22	0.707	
JPT	CC	11	9.64 ± 0.18		0.675	TT	34	9.67 ± 0.20		0.951
	CT	29	9.68 ± 0.19	0.639		TC	9	9.68 ± 0.18	0.859	
	TT	5	9.74 ± 0.25	0.410		CC	2	9.71 ± 0.13	0.785	
	Dominant	34	9.68 ± 0.20	0.549		Dominant	11	9.69 ± 0.17	0.789	
	Recessive	40	9.67 ± 0.19	0.445		Recessive	43	9.67 ± 0.19	0.792	
All	CC	147	9.74 ± 0.19		0.074	TT	84	9.72 ± 0.23		0.087
	CT	108	9.78 ± 0.23	0.121		TC	109	9.77 ± 0.21	0.149	
	TT	15	9.81 ± 0.25	0.212		CC	77	9.79 ± 0.18	**0.029**	
	Dominant	123	9.79 ± 0.23	0.084		Dominant	186	9.78 ± 0.20	**0.040**	
	Recessive	255	9.76 ± 0.21	0.387		Recessive	193	9.75 ± 0.22	0.081	

The significant results were in bold, if the 95% CI excluded 1 or *P* <0.05. *Genotyping data and mRNA expression levels for *XPG* by genotypes were obtained from the HapMap phase II release 23 data from EBV‐transformed lymphoblastoid cell lines from 270 individuals. ^†^Two‐side Student's *t*‐test within the stratum. ^‡^ *P*‐values for the trend test of *XPG* mRNA expression among three genotypes for each SNP from a general linear model.

## Discussion

In the present hospital‐based case–control study, we explored the associations between five potentially functional polymorphisms in the *XPG* gene and the risk of neuroblastoma. We observed significant association between rs2094258 C>T polymorphism and neuroblastoma susceptibility among Southern Chinese children. We found that individuals with rs2094258 CT and CT/TT genotypes were at significantly decreased neuroblastoma risk when compared with those with the CC genotype. We also found that the 2–3 risk genotype carriers had a significantly higher risk of neuroblastoma than the 0–1 risk genotype carriers. The FPRP analysis strengthened the significant associations, while genotype‐based mRNA expression analysis further provided biological evidence for our findings.

The human *XPG* gene is located on chromosome 13q33 and comprises a 30‐kb coding region with 15 exons and 14 introns. This gene encodes a 1186 amino acid structure‐specific endonuclease 33. XPG protein takes part in the transcription‐coupled repair 34, as well as the global genomic NER 35. It also plays an important role in RNA transcription through the interaction with other transcription activator complexes. Improper repair of DNA damage may lead to mutagenesis and cell death 36, 37. The XPG protein can cleave damaged oligonucleotide at the 3′ end. It also cooperates with the XPF/ERCC1 complex that excises damaged oligonucleotide at the 5′ end during NER process. Finally, this protein can stabilize the binding of DNA repair complex to damaged DNA 38, 39, 40. The sequence variations in the DNA repair genes may alter DNA repair capacity, consequentially causing inter‐individual differences in the predisposition to cancer 41. The *XPG* gene is highly polymorphic. According to the dbSNP database (http://www.ncbi.nlm.nih.gov/SNP), there are at least 2432 identified SNPs in the *XPG* gene region, and 654 of them are coding region SNPs. SNPs may lead to the encoded amino acids changes (non‐synonymous), may be silent (synonymous) or may occur in non‐coding regions (transcription factor binding sites or miRNA binding sites). The non‐synonymous SNPs may affect gene function and phenotype and predispose to diseases 42. The most widely investigated SNP in the *XPG* gene is rs17655G>C, which leads aspartate to histidine alteration at codon 1104 24. The association with Asp1104His polymorphism has been widely investigated in different types of cancers, including breast cancer, skin cancer, lung cancer, bladder cancer, head and neck cancer, colorectal cancer and non‐Hodgkin lymphoma 24.

Moreover, three potentially functional *XPG* polymorphisms (rs2094258 C>T, rs2296147 T>C and rs873601G>A) were studied for the association with gastric cancer in a case–control study with 1125 cases and 1196 controls by He *et al*. 26. They found that the rs873601G>A polymorphism (located in the 3′ UTR) was significantly associated with increased gastric cancer risk. They also found that the rs873601 A allele were associated with decreased mRNA expression. Zhu *et al*. 43 also explored the association between these three polymorphisms and oesophageal squamous cell carcinoma (ESCC) susceptibility. They reported a significant association between the rs2296147 C allele and decreased ESCC susceptibility. Duan *et al*. 27 explored the association of rs751402 C>T and rs2296147 T>C polymorphisms with gastric cancer in 403 cases and 403 controls. They found that both of two polymorphisms were associated with an increased gastric cancer risk. Yang *et al*. 44 also genotyped these three polymorphisms in 337 stomach cancer cases and 347 controls. Data showed that the rs2296147 T>C polymorphism was associated with decreased gastric cancer risk, while the rs2094258 C>T polymorphism was associated with increased gastric cancer risk. In a study with 241 prostate cancer cases and 264 controls 45, the rs2296147 T>C polymorphism was shown to associate with increased prostate cancer risk. However, the association with the rs2094258 C>T polymorphism was not replicated in the same study. In the study with 325 breast cancer cases and 325 controls, Na *et al*. 46 tested all of the five polymorphisms included in our study and found significant association between the rs2094258 TT genotype and increased breast cancer risk. Sun *et al*. 47 failed to find any significant association between rs2094258 C>T polymorphism and laryngeal cancer susceptibility with a total of 271 cases and 271 controls.

To the best of our knowledge, this is the first investigation on the association between *XPG* gene polymorphisms and neuroblastoma susceptibility. In this study, we found the rs2094258 C>T polymorphism was associated with decreased neuroblastoma risk. This protective effect of the SNP may be ascribed to the resultant up‐regulation of the *XPG* gene expression. As predicted by SNPinfo (Table S2), the rs2094258 C>T polymorphism is located in the 5′ near region of *XPG* gene and is within a transcription factor binding site. The C>T alteration may lead to an increase in the *XPG* mRNA expression (Table 4) in Chinese subjects as well as in overall individuals. The rs2094258 C>T polymorphism‐induced increase in *XPG* gene expression may enhance an individual's DNA repair capacity, which support the protective association between the rs2094258 T allele and decreased neuroblastoma. Previous studies reported that this polymorphism could increase or decrease cancer risk. The different results may be ascribed to the fact that different cancers may have different environment exposures as well as the difference in sampling in each investigation. Besides, gene–environment interaction may also play important roles in the tumourigenesis 48. It's important to perform FPRP analysis to verify if the significant findings were chance findings or really noteworthy. Moreover, further functional studies are needed to explore the specific mechanisms by which this polymorphism modifies cancer susceptibility.

Though this is the first and largest study to investigate the association of DNA repair gene polymorphisms with neuroblastoma in Chinese children, several limitations in this study should be addressed. First, only 256 cases and 531 controls were included in this study. The relatively small sample size, especially for the cases, may result in limited statistical power. Second, selection bias may exist; since all the subjects were enrolled only from our hospital and restricted to a Chinese Han population, we might miss a larger number of neuroblastoma cases who did not visit our hospital for treatment during the same period. Third, we only included five potentially functional polymorphisms in this study. In the future, all functional SNPs in *XPG* gene should be investigated in different ethnicities, which will yield a meaningful conclusion. Finally, some important information was not available in our study, such as the paternal exposures, living environment and dietary intake for the included children, which limited our ability to perform gene–environmental interactions analysis in neuroblastoma susceptibility.

In conclusion, this study provides evidence that potentially functional polymorphisms in the *XPG* gene, especially the rs2094258 C>T polymorphism, may contribute to neuroblastoma susceptibility in Southern Chinese children. However, further prospective studies with larger sample size involving different ethnicities, as well as further functional studies, are needed to confirm our findings.

## Conflicts of interest

The authors declare no competing financial interests.

## Supporting information


**Table S1** Frequency distribution of selected characteristics in neuroblastoma patients and controls.
**Table S2** Potential function of the five selected SNPs in *XPG* gene as predicted by SNPinfo software.

## References

[1] Smith MA , Seibel NL , Altekruse SF , *et al* Outcomes for children and adolescents with cancer: challenges for the twenty‐first century. J Clin Oncol. 2010; 28: 2625–34. 20404250 10.1200/JCO.2009.27.0421 PMC2881732

[2] London WB , Castleberry RP , Matthay KK , *et al* Evidence for an age cutoff greater than 365 days for neuroblastoma risk group stratification in the Children's Oncology Group. J Clin Oncol. 2005; 23: 6459–65. 16116153 10.1200/JCO.2005.05.571

[3] Matthay KK , Villablanca JG , Seeger RC , *et al* Treatment of high‐risk neuroblastoma with intensive chemotherapy, radiotherapy, autologous bone marrow transplantation, and 13‐cis‐retinoic acid. Children's Cancer Group. N Engl J Med. 1999; 341: 1165–73. 10519894 10.1056/NEJM199910143411601

[4] Bao PP , Li K , Wu CX , *et al* Recent incidences and trends of childhood malignant solid tumors in Shanghai, 2002–2010. Zhonghua Er Ke Za Zhi. 2013; 51: 288–94. 23927803

[5] De Roos AJ , Olshan AF , Teschke K , *et al* Parental occupational exposures to chemicals and incidence of neuroblastoma in offspring. Am J Epidemiol. 2001; 154: 106–14. 11447042 10.1093/aje/154.2.106

[6] De Roos AJ , Teschke K , Savitz DA , *et al* Parental occupational exposures to electromagnetic fields and radiation and the incidence of neuroblastoma in offspring. Epidemiology. 2001; 12: 508–17. 11505168 10.1097/00001648-200109000-00008

[7] Maris JM , Mosse YP , Bradfield JP , *et al* Chromosome 6p22 locus associated with clinically aggressive neuroblastoma. N Engl J Med. 2008; 358: 2585–93. 18463370 10.1056/NEJMoa0708698 PMC2742373

[8] Capasso M , Devoto M , Hou C , *et al* Common variations in BARD1 influence susceptibility to high‐risk neuroblastoma. Nat Genet. 2009; 41: 718–23. 19412175 10.1038/ng.374 PMC2753610

[9] le Nguyen B , Diskin SJ , Capasso M , *et al* Phenotype restricted genome‐wide association study using a gene‐centric approach identifies three low‐risk neuroblastoma susceptibility Loci. PLoS Genet. 2011; 7: e1002026. 21436895 10.1371/journal.pgen.1002026 PMC3060064

[10] Wang K , Diskin SJ , Zhang H , *et al* Integrative genomics identifies LMO1 as a neuroblastoma oncogene. Nature. 2011; 469: 216–20. 21124317 10.1038/nature09609 PMC3320515

[11] Diskin SJ , Capasso M , Schnepp RW , *et al* Common variation at 6q16 within HACE1 and LIN28B influences susceptibility to neuroblastoma. Nat Genet. 2012; 44: 1126–30. 22941191 10.1038/ng.2387 PMC3459292

[12] Latorre V , Diskin SJ , Diamond MA , *et al* Replication of neuroblastoma SNP association at the BARD1 locus in African‐Americans. Cancer Epidemiol Biomarkers Prev. 2012; 21: 658–63. 22328350 10.1158/1055-9965.EPI-11-0830 PMC3319325

[13] Capasso M , Diskin SJ , Totaro F , *et al* Replication of GWAS‐identified neuroblastoma risk loci strengthens the role of BARD1 and affirms the cumulative effect of genetic variations on disease susceptibility. Carcinogenesis. 2013; 34: 605–11. 23222812 10.1093/carcin/bgs380 PMC3716226

[14] Lu J , Chu P , Wang H , *et al* Candidate gene association analysis of neuroblastoma in chinese children strengthens the role of LMO1. PLoS ONE. 2015; 10: e0127856. 26030754 10.1371/journal.pone.0127856 PMC4452511

[15] He J , Zhang R , Zou Y , *et al* Evaluation of GWAS‐identified SNPs at 6p22 with neuroblastoma susceptibility in a Chinese population. Tumour Biol. 2015; doi:10.1007/s13277‐015‐3936‐7. 10.1007/s13277-015-3936-7 26307394

[16] Frazer KA , Murray SS , Schork NJ , *et al* Human genetic variation and its contribution to complex traits. Nat Rev Genet. 2009; 10: 241–51. 19293820 10.1038/nrg2554

[17] Stadler ZK , Thom P , Robson ME , *et al* Genome‐wide association studies of cancer. J Clin Oncol. 2010; 28: 4255–67. 20585100 10.1200/JCO.2009.25.7816 PMC2953976

[18] Han W , Zhou Y , Zhong R , *et al* Functional polymorphisms in FAS/FASL system increase the risk of neuroblastoma in Chinese population. PLoS ONE. 2013; 8: e71656. 23951214 10.1371/journal.pone.0071656 PMC3741122

[19] Jin Y , Wang H , Han W , *et al* Single nucleotide polymorphism rs11669203 in TGFBR3L is associated with the risk of neuroblastoma in a Chinese population. Tumour Biol. 2015; doi:10.1007/s13277‐015‐4192‐6. 10.1007/s13277-015-4192-6 26468016

[20] Wood RD , Mitchell M , Sgouros J , *et al* Human DNA repair genes. Science. 2001; 291: 1284–9. 11181991 10.1126/science.1056154

[21] Friedberg EC . How nucleotide excision repair protects against cancer. Nat Rev Cancer. 2001; 1: 22–33. 11900249 10.1038/35094000

[22] Cleaver JE . Common pathways for ultraviolet skin carcinogenesis in the repair and replication defective groups of xeroderma pigmentosum. J Dermatol Sci. 2000; 23: 1–11. 10699759 10.1016/s0923-1811(99)00088-2

[23] Clarkson SG . The XPG story. Biochimie. 2003; 85: 1113–21. 14726017 10.1016/j.biochi.2003.10.014

[24] Zhu ML , Wang M , Cao ZG , *et al* Association between the ERCC5 Asp1104His polymorphism and cancer risk: a meta‐analysis. PLoS ONE. 2012; 7: e36293. 22815677 10.1371/journal.pone.0036293 PMC3399856

[25] Zhang R , Zou Y , Zhu J , *et al* The association between GWAS‐identified BARD1 gene SNPs and neuroblastoma susceptibility in a Southern Chinese population. Int J Med Sci. 2016; 13: 133–8. 26941572 10.7150/ijms.13426 PMC4764780

[26] He J , Qiu LX , Wang MY , *et al* Polymorphisms in the XPG gene and risk of gastric cancer in Chinese populations. Hum Genet. 2012; 131: 1235–44. 22371296 10.1007/s00439-012-1152-8

[27] Duan Z , He C , Gong Y , *et al* Promoter polymorphisms in DNA repair gene ERCC5 and susceptibility to gastric cancer in Chinese. Gene. 2012; 511: 274–9. 22982416 10.1016/j.gene.2012.09.025

[28] He J , Liao XY , Zhu JH , *et al* Association of MTHFR C677T and A1298C polymorphisms with non‐Hodgkin lymphoma susceptibility: evidence from a meta‐analysis. Sci Rep. 2014; 4: 6159. 25146845 10.1038/srep06159 PMC5381410

[29] Shi TY , He J , Wang MY , *et al* CASP7 variants modify susceptibility to cervical cancer in Chinese women. Sci Rep. 2015; 5: 9225. 25784056 10.1038/srep09225 PMC4363885

[30] Wacholder S , Chanock S , Garcia‐Closas M , *et al* Assessing the probability that a positive report is false: an approach for molecular epidemiology studies. J Natl Cancer Inst. 2004; 96: 434–42. 15026468 10.1093/jnci/djh075 PMC7713993

[31] He J , Wang MY , Qiu LX , *et al* Genetic variations of mTORC1 genes and risk of gastric cancer in an Eastern Chinese population. Mol Carcinog. 2013; 52: E70–9. 23423739 10.1002/mc.22013

[32] Brodeur GM , Pritchard J , Berthold F , *et al* Revisions of the international criteria for neuroblastoma diagnosis, staging, and response to treatment. J Clin Oncol. 1993; 11: 1466–77. 8336186 10.1200/JCO.1993.11.8.1466

[33] Emmert S , Schneider TD , Khan SG , *et al* The human XPG gene: gene architecture, alternative splicing and single nucleotide polymorphisms. Nucleic Acids Res. 2001; 29: 1443–52. 11266544 10.1093/nar/29.7.1443 PMC31292

[34] Le Page F , Kwoh EE , Avrutskaya A , *et al* Transcription‐coupled repair of 8‐oxoguanine: requirement for XPG, TFIIH, and CSB and implications for Cockayne syndrome. Cell. 2000; 101: 159–71. 10786832 10.1016/s0092-8674(00)80827-2

[35] Hanawalt PC . Controlling the efficiency of excision repair. Mutat Res. 2001; 485: 3–13. 11341989 10.1016/s0921-8777(00)00071-9

[36] Lee SK , Yu SL , Prakash L , *et al* Requirement of yeast RAD2, a homolog of human XPG gene, for efficient RNA polymerase II transcription. implications for Cockayne syndrome. Cell. 2002; 109: 823–34. 12110180 10.1016/s0092-8674(02)00795-x

[37] Barreto G , Schafer A , Marhold J , *et al* Gadd45a promotes epigenetic gene activation by repair‐mediated DNA demethylation. Nature. 2007; 445: 671–5. 17268471 10.1038/nature05515

[38] Wakasugi M , Reardon JT , Sancar A . The non‐catalytic function of XPG protein during dual incision in human nucleotide excision repair. J Biol Chem. 1997; 272: 16030–4. 9188507 10.1074/jbc.272.25.16030

[39] O'Donovan A , Davies AA , Moggs JG , *et al* XPG endonuclease makes the 3′ incision in human DNA nucleotide excision repair. Nature. 1994; 371: 432–5. 8090225 10.1038/371432a0

[40] Friedberg EC . DNA damage and repair. Nature. 2003; 421: 436–40. 12540918 10.1038/nature01408

[41] Chen Z , Yang J , Wang G , *et al* Attenuated expression of xeroderma pigmentosum group C is associated with critical events in human bladder cancer carcinogenesis and progression. Cancer Res. 2007; 67: 4578–85. 17510383 10.1158/0008-5472.CAN-06-0877

[42] Shastry BS . SNPs: impact on gene function and phenotype. Methods Mol Biol. 2009; 578: 3–22. 19768584 10.1007/978-1-60327-411-1_1

[43] Zhu ML , Shi TY , Hu HC , *et al* Polymorphisms in the ERCC5 gene and risk of esophageal squamous cell carcinoma (ESCC) in Eastern Chinese populations. PLoS ONE. 2012; 7: e41500. 22848513 10.1371/journal.pone.0041500 PMC3406052

[44] Yang WG , Zhang SF , Chen JW , *et al* SNPs of excision repair cross complementing group 5 and gastric cancer risk in Chinese populations. Asian Pac J Cancer Prev. 2012; 13: 6269–72. 23464443 10.7314/apjcp.2012.13.12.6269

[45] Yang B , Chen WH , Wen XF , *et al* Role of DNA repair‐related gene polymorphisms in susceptibility to risk of prostate cancer. Asian Pac J Cancer Prev. 2013; 14: 5839–42. 24289586 10.7314/apjcp.2013.14.10.5839

[46] Na N , Dun E , Ren L , *et al* Association between ERCC5 gene polymorphisms and breast cancer risk. Int J Clin Exp Pathol. 2015; 8: 3192–7. 26045839 PMC4440148

[47] Sun Y , Tan L , Li H , *et al* Association of NER pathway gene polymorphisms with susceptibility to laryngeal cancer in a Chinese population. Int J Clin Exp Pathol. 2015; 8: 11615–21. 26617899 PMC4637715

[48] Zhu B , Tian J , Zhong R , *et al* Genetic variants in the SWI/SNF complex and smoking collaborate to modify the risk of pancreatic cancer in a Chinese population. Mol Carcinog. 2015; 54: 761–8. 24585446 10.1002/mc.22140

