# Supplementary material for: Association of potentially functional variants in the XPG gene with neuroblastoma risk in a Chinese population
Source: J Cell Mol Med. 2016 Mar 28;20(8):1481–90. doi: 10.1111/jcmm.12836 (PMC4956948; doi:10.1111/jcmm.12836)
Supplement: Supplementary file 1 — Table S1 Frequency distribution of selected characteristics in neuroblastoma patients and controls. Table S2 Potential function of the five selected SNPs in XPG gene as predicted by SNPinfo software. [file JCMM-20-1481-s001.doc]

| **Supplemental Table 1.** Frequency distribution of selected characteristics in neuroblastoma patients and controls | | | | | |
| --- | --- | --- | --- | --- | --- |
| Variables | Cases (n=256) | | Controls (n=531) | | *P^a^* |
|  | No. | % | No. | % |  |
| Age range, month | 0-156 | | 0.07-156 | | 0.239 |
| Mean ± SD | 30.87 ± 26.45 | | 29.73 ± 24.86 | |  |
| ≤18 | 101 | 39.45 | 233 | 43.88 |  |
| >18 | 155 | 60.55 | 298 | 56.12 |  |
| Gender |  |  |  |  | 0.333 |
| Female | 103 | 40.23 | 233 | 43.88 |  |
| Male | 153 | 59.77 | 298 | 56.12 |  |
| Clinical stage |  |  |  |  |  |
| I | 54 | 21.09 |  |  |  |
| II | 65 | 25.39 |  |  |  |
| III | 44 | 17.19 |  |  |  |
| IV | 77 | 30.08 |  |  |  |
| 4s | 9 | 3.52 |  |  |  |
| NA | 7 | 2.73 |  |  |  |
| Site of origin |  |  |  |  |  |
| Adrenal gland | 46 | 17.97 |  |  |  |
| Retroperitoneal region | 87 | 33.98 |  |  |  |
| Mediastinum | 90 | 35.16 |  |  |  |
| Other region | 25 | 9.77 |  |  |  |
| NA | 8 | 3.13 |  |  |  |
| ^a^ Two-sided *χ^2^* test for distributions between neuroblastoma patients and controls | | | | | |

| **Supplemental Table 2. Potential function of the five selected SNPs in *XPG* gene as predicted by SNPinfo software** | | | | | | | | | | | | | |
| --- | --- | --- | --- | --- | --- | --- | --- | --- | --- | --- | --- | --- | --- |
| **rs** | **Chr** | **Allele** | **Position** | **Location** | **TFBS** | **Splicing**  **(ESE or ESS)** | **miRNA**  **(miRanda)** | **miRNA**  **(Sanger)** | **Nearby Gene** | **Distance (bp)** | **Allele** | **Asian** | **CHB** |
| rs2094258 | 13 | C/T | 102294760 | 5' near gene | Y | -- | -- | -- | BIVM||ERCC5 | -2878||-1415 | C | 0.627 | 0.661 |
| rs751402 | 13 | C/T | 102296199 | 5' UTR | Y | Y | -- | -- | ERCC5 | 24||30147 | C | 0.591 | 0.619 |
| rs2296147 | 13 | T/C | 102296376 | 5' UTR | Y | -- | -- | -- | ERCC5 | 201||29970 | T | 0.838 | 0.768 |
| rs1047768 | 13 | T/C | 102302518 | exon | -- | Y | -- | -- | ERCC5 | 6343||23828 | T | 0.778 | 0.720 |
| rs873601 | 13 | G/A | 102326338 | 3' UTR | -- | Y | Y | Y | ERCC5 | 30163||8 | G | 0.517 | 0.464 |
| SNP, single nucleotide polymorphism; XPG, xeroderma pigmentosum group G; TFBS, transcription factor binding site; ESE, exon splicing enhancer; ESS, exon splicing silencer; CHB, Han Chinese in Beijing, China; UTR, untranslated region; ERCC5, excision repair cross-complementation group 5 | | | | | | | | | | | | | |
